# Th1 and Th17 Responses to LTB and Colonization Factors Following Oral ETEC Vaccination

**DOI:** 10.3390/microorganisms14092007

**Published:** 2026-09-10

**Authors:** Joanna Kaim, Anna Lundgren

**Affiliations:** GUVAX (Gothenburg University Vaccine Research Institute), Department of Microbiology and Immunology, Institute of Biomedicine, University of Gothenburg, 405 30 Gothenburg, Sweden; joanna.kaim@microbio.gu.se

**Keywords:** ETEC, vaccine, T cell, cytokine, dmLT, adjuvant

## Abstract

T helper cells (Th) are central to mucosal IgA induction and key targets for modulation by vaccine adjuvants. To improve understanding of cellular mechanisms underlying mucosal vaccine-induced immunity, we analyzed antigen-specific peripheral blood Th responses elicited by the oral enterotoxigenic *Escherichia coli* (ETEC) vaccine ETVAX, administered with or without the double mutant heat-labile toxin (dmLT) adjuvant. ETVAX, consisting of inactivated *E. coli* overexpressing colonization factors CFA/I, CS3, CS5, and CS6 with a heat-labile toxin B-subunit toxoid, was given orally in two doses to adult volunteers, either alone or with 10 or 25 µg dmLT. Antigen-specific Th-associated cytokine responses were assessed in stimulated peripheral blood mononuclear cells isolated from 15 to 18 individuals/group using ELISA and electrochemiluminescence assays. ETVAX predominantly induced Th1 (IFN-γ) and Th17 (IL-17A) responses, with minimal Th2-associated cytokines. Responses were markedly reduced after CD4+ T-cell depletion, supporting a Th cell origin. The strongest responses targeted LTB and CS3, with IFN-γ responses detected in 60–80% and IL-17A in 40–60% across all vaccinees. Responses to CFA/I, CS5 and CS6 were generally weaker. Exploratory comparisons suggested broader IFN-γ responses and more consistent IFN-γ and IL-17A responses to lower-dose antigens, particularly CS6, in recipients receiving vaccine plus 10 µg dmLT. These trends paralleled IgA antibody-secreting cell response patterns, with significantly enhanced IgA responses to CS6 in the vaccine plus 10 µg dmLT group. In conclusion, ETVAX induces antigen-specific Th1- and Th17-type responses in peripheral blood, supporting a role for cellular immunity in mucosal responses to oral ETEC vaccines.

## 1. Introduction

Enterotoxigenic *Escherichia coli* (ETEC) is a leading bacterial cause of diarrheal morbidity and mortality among infants and young children in low- and middle-income countries [1,2,3]. ETEC is also the most common cause of diarrhea in travelers [4]. Despite this substantial global disease burden, no licensed ETEC vaccine is currently available. Protective immunity against ETEC is considered to be mediated primarily by the synergistic action of intestinal IgA antibodies targeting the colonization factors (CFs) on the bacterial surface, which bind to the intestinal epithelial cells, and the heat-labile toxin (LT), which causes watery diarrhea [1,5,6]. Human trials of oral inactivated or attenuated ETEC vaccines have demonstrated robust intestinal IgA responses against both CFs and LT B-subunit (LTB) [6,7,8]. However, the cellular mechanisms underlying the induction and maintenance of these mucosal antibody responses remain incompletely understood.

CD4+ T helper (Th) cells are likely to be essential for primary B-cell activation, affinity maturation and the development of durable memory B-cell responses to ETEC antigens. In addition to their mechanistic role, antigen-specific T-cell responses may serve as useful biomarkers of vaccine take, priming efficiency, adjuvant action and immune memory in clinical vaccine trials [9]. Nevertheless, detailed analyses of CF- and LTB-specific Th-cell responses following oral ETEC vaccination in humans remain limited.

The oral vaccine candidate ETVAX is currently the most advanced ETEC vaccine candidate and has undergone extensive Phase I/II evaluation in adults and children [10,11,12,13]. This vaccine consists of four inactivated recombinant *E. coli* strains overexpressing the most prevalent CFs (CFA/I, CS3, CS5 and CS6) and is co-administered with the LT-derived hybrid toxoid LCTBA [14,15]. The vaccine has been tested with and without the double mutant LT (dmLT) adjuvant in multiple studies, including adults, young children and infants [11,12,13,16]. The vaccine induces significant fecal and intestine-derived antibody-secreting cell (ASC) responses in peripheral blood to all CFs in the vaccine and LTB in Swedish adults, with immunological memory persisting for at least 1–2 years after primary vaccinations [15,17]. Addition of dmLT significantly enhanced primary IgA ASC responses to CS6, which is the antigen present in the lowest amounts in the vaccine [15]. Robust mucosal and systemic IgA responses to ETVAX with dmLT have also been demonstrated in infants, children and adults in ETEC endemic settings in Bangladesh, Zambia and the Gambia [12,13,18,19]. Notably, ETVAX has recently been shown to confer significant protection against ETEC-associated diarrhea in Gambian infants [13]. Oral ETVAX vaccination induces activation of circulating T follicular helper (cTfh)-like cells in adults, suggesting engagement of T-cell dependent B-cell responses [20]. However, antigen-specific Th-cell responses induced by ETVAX and other ETEC vaccine candidates remain poorly characterized.

Cholera toxin (CT), LT and their detoxified derivatives are potent mucosal adjuvants with the capacity to enhance antigen-presenting cell (APC) function and modulate T-cell responses [16]. Modified LT and CT molecules, including single mutant LT (mLT), dmLT and multiple mutant CT (mmCT), exhibit reduced or abolished toxicity while retaining adjuvant function, and have been shown to modulate cytokine expression in both APCs and T cells [16,21,22,23]. We and others have shown that these adjuvants preferentially promote Th17-type responses in human immune cells in vitro [21,22,24] and in vivo mouse vaccination models [16,25,26,27]. Whether dmLT similarly modulates antigen-specific human T-cell responses in vivo following oral vaccination has not been established.

In this study, we used samples from a previously reported clinical trial of ETVAX administered to healthy Swedish adults to characterize the antigen specificity and cytokine profile of vaccine-induced T-cell responses [15]. We further examined the relationship between T-cell responses and intestine-derived IgA ASC responses to gain insight into the cellular mechanisms underlying mucosal antibody induction and the potential use of T-cell responses as biomarkers of vaccine-induced immunity. We also explored whether response patterns differed between subjects receiving ETVAX with or without dmLT.

## 2. Materials and Methods

### 2.1. Vaccine, Participants, and Samples

A detailed description of the ETVAX vaccine, original vaccine trial design and primary endpoint analyses has been reported previously [15]. Briefly, ETVAX consists of four inactivated recombinant *E. coli* strains which overexpress CFA/I, CS3, CS5 and CS6, respectively, combined with the LTB/cholera toxin B-subunit (CTB) hybrid protein LCTBA [14,15,28,29]. dmLT (R192G/L211A) is an LT-derived protein containing two genetic substitutions in the A subunit that eliminate the enterotoxic activity while preserving adjuvant activity [30].

In the original trial, Swedish adults received two oral doses of ETVAX alone (*n* = 35), vaccine + 10 µg dmLT (*n* = 30), vaccine + 25 µg dmLT (*n* = 30) or placebo (*n* = 34; buffer alone) [15]. Primary endpoints of the original trial were safety and antibody responses to the five primary vaccine components (CFA/I, CS3, CS5, CS6 and LTB). dmLT effects on antibody responses were also explored. Vaccine-induced T-cell responses were included as exploratory endpoints of the trial.

Heparinized blood was collected prior to vaccination (day 0), 7 days after the first dose, and 5 and 7 days after the second dose (corresponding to days 19 and 21 after the first dose). Subjects from the three vaccination groups were randomly selected for inclusion in the present T-cell study based solely on the availability of cryopreserved peripheral blood mononuclear cells (PBMCs), with 17–18 subjects included per vaccine group (Table 1). No immunological or clinical outcome measures were considered during subject selection. The only exception was a small number of subjects used for assay optimization, who were selected based on their ASC responses to different vaccine antigens, as measured by the antibodies in lymphocyte supernatants (ALS) assay.

The original trial and this T-cell sub-study were conducted in accordance with the Declaration of Helsinki and approved by the Ethical Committee in the Gothenburg Region (reg no 946-11) and the Western Institutional Review Board, USA (reg no 20112026). Written informed consent was obtained from all participants. The vaccine trial is registered in the EudraCT (2011-003228-11) and ISCRTN databases (ISRCTN9136076).

### 2.2. T-Cell Analysis

PBMCs were isolated by density centrifugation of Ficoll-Paque, cryopreserved according to the protocol established by Kreher et al. and used for stimulation experiments after thawing [31]. Cell viability exceeded 95%, as assessed by live/dead flow cytometric staining and trypan blue exclusion. CD4+ T cells were depleted from PBMCs using Dynabeads (Invitrogen, Waltham, MA, USA) according to the instructions provided by the manufacturer.

PBMCs and CD4+ T-cell depleted PBMCs (1.5 × 10^5^ cells/well, 200 µL/well) were stimulated with LTB (10 µg/mL, Scandinavian Biopharma, Solna, Sweden), CFA/I (1 µg/mL, produced at University of Gothenburg, Gothenburg, Sweden), CS3 (1 µg/mL, Scandinavian Biopharma, Sweden), CS5 (1 µg/mL, Scandinavian Biopharma) or CS6 (1 µg/mL, kindly provided by F. Cassel, National Institutes of Health, Bethesda, MD, USA). Due to limited cell availability, PBMCs from 1 to 2 subjects per group were stimulated with only a subset (2–4) of antigens, resulting in 15–18 analyzed samples per group and antigen. Cells were cultured in 96-well U-bottomed culture plates (Nunc, Thermo Fisher Scientific, Waltham, MA, USA) in DMEM/F12 medium (Invitrogen) supplemented with 5% human serum and 1% gentamicin at 37 °C in 5% CO_2_. Supernatants (150 µL per well) were collected after 48 h (setup experiments) or after 5 days (all experiments) and stored at −70 °C until cytokine analyses.

In assay optimization experiments, cytokines (IFN-γ, IL-17A, IL-4, IL-5, IL-13, IL-10, IL-6, IL-8, TNF, IL-1β, IL-2 and IL-12p40) were analyzed in cell culture supernatants by multiplex electrochemiluminescence (ECL) assays (Meso Scale Discovery, Rockville, MD, USA). In the full T-cell analysis, IFN-γ was quantified by ELISA (IFN-γ, eBioscience, assay range 8–500 pg/mL), whereas IL-17A was quantified using the more sensitive ECL platform (assay range 1.4–5670 pg/mL), as preliminary analyses demonstrated substantially lower IL-17A levels in stimulated PBMC cultures. All assays were performed according to the instructions provided by the manufacturers and positive controls were included in all assay runs to ensure reproducibility.

For analysis of the purity of PBMCs depleted of CD4+ T cells, PBMCs were stained with combinations of the following antibodies: anti-CD14 FITC, anti-CD4 PerCP, anti-CD19 PE, anti-CD3 APC and anti-CD8 FITC (BD Biosciences, Franklin Lakes, NJ, USA). Cells were analyzed using LSR II (BD Biosciences, Franklin Lakes, NJ, USA) and the data was analyzed with FlowJo software (Tree Star Inc., Ashland, OR, USA, Version 10). The frequency of CD4+ cells in PBMCs after depletion of CD3+CD4+ cells was <5%.

### 2.3. Antibody Analysis

Previously published antigen-specific ASC responses measured by the ALS assay in the original trial were included to assess the representativeness of the T-cell analysis subset relative to the full study population and to examine associations between cellular and humoral immune responses [15]. ASCs induced by oral vaccination express the gut homing marker integrin α4β7 and correlate with ASC responses in intestinal mucosa as well as with secretory IgA (SIgA) levels in fecal and intestinal lavage samples, supporting their use as surrogate markers of mucosal immune responses [32,33,34,35,36,37]. In the ALS assay, antibodies secreted ex vivo by circulating plasma blasts/plasma cells (i.e., ASCs) are quantified in culture supernatants and provide results comparable to those obtained by the more traditional ELISPOT assay for assessment of intestine-derived ASC responses following oral vaccination [15,29,38,39,40].

Briefly, freshly isolated PBMCs (2 × 10^6^ cells per well) were cultured in 96-well plates and supernatants were collected after 72 h [15,29]. Antigen-specific IgA levels in ALS specimens were analyzed by ELISA using plates coated with CFA/I, CS3, CS5 or CS6 or with GM1 plus LTB [15,29].

### 2.4. Statistical Analysis

Data was evaluated using GraphPad Prism (Version 11, GraphPad Software, Inc., San Diego, CA, USA). Statistical differences between pre- and post-vaccination cytokine and IgA antibody levels were evaluated using the Wilcoxon matched-pairs signed-rank test. Differences in cytokine responses between vaccine groups were evaluated using the Mann–Whitney test. Differences in ALS IgA levels between groups were evaluated using a *t*-test after log transformation of the data, as performed in the original study [15]. Correlations were assessed using Spearman’s rank correlation test.

Response magnitudes were expressed as fold increases, defined as the maximum cytokine or antibody level detected at any post-vaccination time point (7 days after the first dose or 5 or 7 days after the second dose) divided by the corresponding pre-vaccination level. Subjects with fold-increases ≥ 2 were classified as responders across all assays. Differences in responder frequencies between groups were analyzed using Fisher’s exact test.

*p*-values < 0.05 were considered statistically significant. To maintain consistency with the original study publication [15], comparisons of ALS responses between the vaccine alone and vaccine + dmLT groups in the T-cell analysis subset were analyzed with and without Holm’s correction for multiple testing. As all T-cell analyses were exploratory, only included a subset of the original study population and intended primarily to identify response patterns and generate hypotheses for future studies, no formal adjustment for multiple comparisons was applied. Accordingly, statistically significant findings should be interpreted with caution.

## 3. Results

### 3.1. Establishment of a Protocol for Analysis of ETVAX-Induced T-Cell Responses

**Figure 1:**
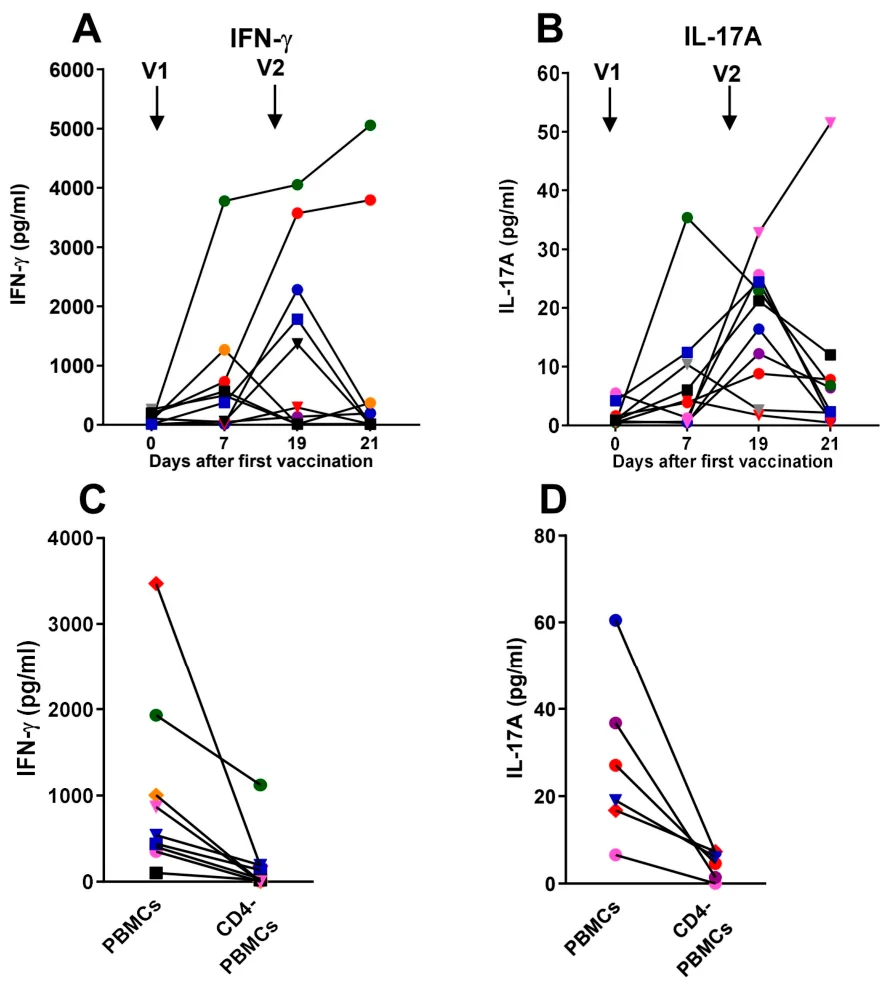
Kinetics and cellular origin of IFN-γ and IL-17A responses to LTB and colonization factors following oral ETVAX vaccination. IFN-γ (**A**,**C**) and IL-17A (**B**,**D**) concentrations in culture supernatants from PBMCs stimulated with LTB, CFA/I, CS3 (only **C**,**D**) or CS6. (**A**,**B**) Responses were analyzed before vaccination (day 0), 7 days after the first vaccine dose (V1) and 5 and 7 days after the second dose (V2), corresponding to 19 or 21 days after the first vaccination, in eight participants. For each antigen, only subjects exhibiting ≥ 2-fold increase in cytokine production in at least one post-vaccination sample relative to baseline are shown (LTB, *n* = 5; CFA/I, *n* = 2; CS6, *n* = 3 for both IFN-γ and IL-17A). (**C**,**D**) Cytokine responses in total PBMCs and in CD4+ T cell-depleted PBMCs (CD4- PBMCs) were compared on day 5 after the second vaccine dose, corresponding to 19 days after the first dose, in seven participants, of whom one was also included in (**A**,**B**). For each antigen, only subjects exhibiting ≥ 2-fold increase in cytokine production in the day 19 sample relative to baseline are shown (IFN-γ: LTB, *n* = 3; CFA/I, *n* = 2; CS3, *n* = 2; CS6, n = 2; IL-17A: LTB, *n* = 4; CS3, *n* = 1; CS6, *n* = 1). (**A**–**D**) Colors denote individual participants (consistent between **A**,**B** and between **C**,**D**), and symbols indicate the stimulating antigen (LTB, circles; CFA/I, squares; CS3, diamonds; CS6, triangles). Cytokine levels in unstimulated cultures were subtracted from all values shown.

To develop a protocol for assessing antigen-specific T-cell responses after oral ETEC vaccination, response kinetics and cytokine profiles were analyzed in a subset of individuals (*n* = 8) previously shown to respond strongly in the IgA ALS assay to multiple ETVAX antigens [15]. PBMCs isolated before and after vaccination were stimulated with LTB or CFs and supernatants were screened for cytokines using highly sensitive ECL multiplex assays. Vaccination induced consistent increases in IFN-γ and IL-17A production in response to stimulation with both LTB and CFs (Figure 1A,B). In contrast, levels of IL-4, IL-5, IL-13, IL-10, IL-6, IL-8, TNF, IL-1β, IL-2 and IL-12p40 remained below or near detection limits or did not change with vaccination.

IFN-γ responses were detected after both 2 and 5 days of PBMC stimulation, whereas robust IL-17A responses were primarily observed after 5 days. Although response kinetics varied between individuals, the highest IFN-γ and IL-17A levels were most commonly detected 7 days after the first dose or 5 days after the second (i.e., 19 days after the first dose) (Figure 1A,B). Peak responses were only rarely observed 7 days after the second dose and mainly in subjects also responding at previous time points. IFN-γ was produced at substantially higher levels than IL-17A and was detectable by both ELISA and ECL in response to all five antigens. In contrast, IL-17A responses to all antigens except CS5 were consistently detected by ECL, but not by ELISA.

To identify the cellular source of IFN-γ and IL-17A, responses were compared in intact PBMCs and PBMCs depleted of CD4+ T cells. Cytokine production induced by LTB or CF stimulation was almost completely abolished following CD4+ T cell depletion in all but one subject, indicating that the responses were primarily derived from CD4+ Th cells (Figure 1C,D).

Based on these findings, a standardized T-cell analysis protocol was established using 5-day antigen stimulation followed by measurement of IFN-γ and IL-17A in culture supernatants. IFN-γ responses to all five vaccine antigens were analyzed by ELISA at all four sampling time points, while IL-17A responses were analyzed by ECL at the first three time points. A ≥ 2-fold increase over baseline was used to define a positive response, consistent with previous analyses of vaccine-specific IgA ALS responses [15].

### 3.2. Representativeness of the T-Cell Analysis Subset

Participants with sufficient numbers of cryopreserved PBMCs to complete the established analysis protocol were included in the T-cell analyses (*n* = 17–18 per group). The demographic characteristics are summarized in Table 1. Overall, the included participants were comparable to the original study population. The largest difference was observed in the vaccine + 25 µg dmLT group, where males constituted 89% of the subset compared with 73% of the corresponding total study population.

To further assess the representativeness of the subset, previously reported vaccine-induced ALS IgA responses to the major vaccine antigens were compared between the subset and the original study cohort [15]. Response magnitudes were broadly comparable between the subset and the total study population, whether all vaccine recipients were analyzed together (Supplementary Figure S1) or stratified by vaccination group (Supplementary Figure S2). In both populations, the strongest ALS IgA responses were directed against LTB and CS3, although significant responses to CFA/I, CS5 and CS6 were also detected (Supplementary Figure S1). When analyses were performed within individual vaccine groups, significantly enhanced IgA response magnitudes to CS6 were observed in the low-dose dmLT group compared to vaccine alone in the total study population, and a similar trend was seen for CS5 (Supplementary Figure S2D,E). In the T-cell subset, these differences reached statistical significance for both CS5 and CS6.

Taken together, these findings indicate that the subset was representative of the total study population and was therefore suitable for characterization of vaccine-induced T-cell responses.

### 3.3. Antigen Specificity, Magnitude and Breadth of Vaccine-Induced T-Cell Responses

**Figure 2:**
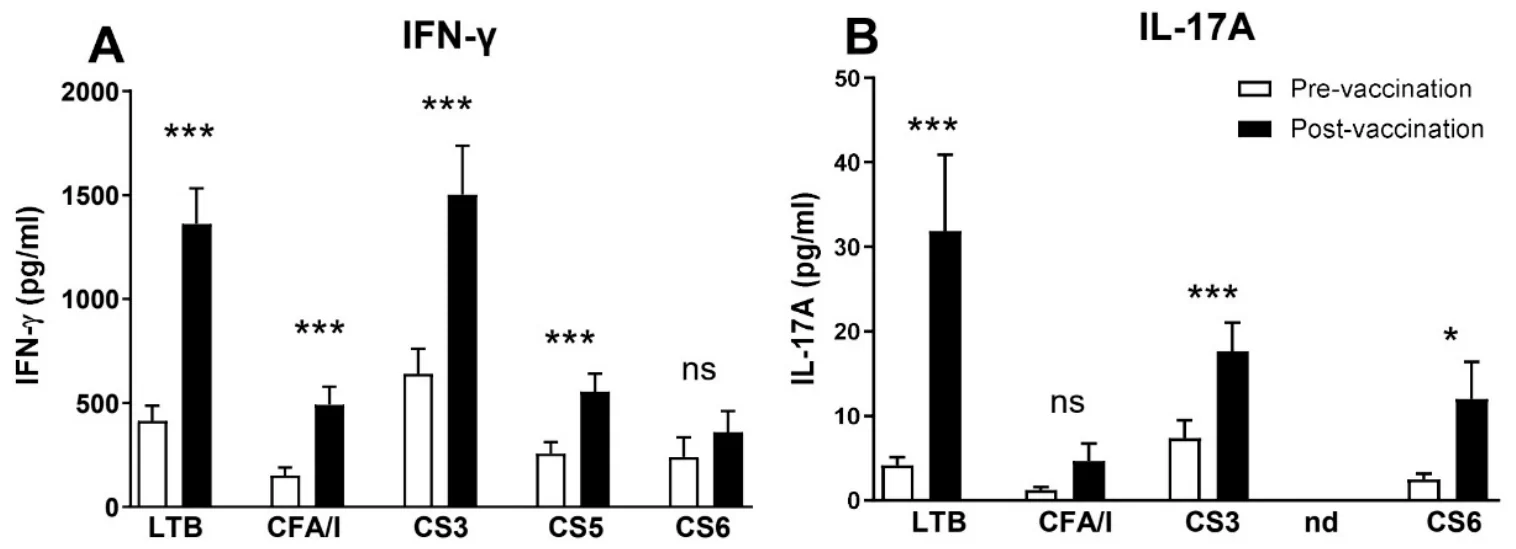
ETVAX-induced IFN-γ and IL-17A responses to LTB and colonization factors in all vaccination groups combined. IFN-γ (**A**) and IL-17A (**B**) concentrations were measured in culture supernatants from PBMCs stimulated with LTB, CFA/I, CS3, CS5 (IFN-γ only) and CS6 before and after vaccination. All subjects with available cryopreserved cells from all three vaccination groups were pooled (total *n* = 47–52, *n* = 15–18 per group and antigen). Results are presented as mean + SEM of the maximal concentrations of cytokines detected at any post-vaccination time point (black bars) compared to pre-vaccination levels (white bars). Cytokine levels in unstimulated cultures were subtracted from all values shown. * *p* < 0.05, *** *p* < 0.001; ns, *p* > 0.05; post- versus pre-vaccination, nd; not determined.

T-cell responses were first evaluated across all vaccinees irrespective of vaccination group. Similar to the ALS IgA responses, the strongest IFN-γ and IL-17A responses were directed against LTB and CS3 (Figure 2). IFN-γ responses were significantly increased for all antigens except CS6, while IL-17A responses were significantly increased for LTB, CS3, and CS6, but not CFA/I (IL-17A not analyzed for CS5). Responder frequencies broadly mirrored response magnitudes (Table 2), with the highest proportions of IFN-γ and IL-17A responders observed for LTB (60–70%). For IFN-γ, responder frequencies were also relatively high for CFA/I and CS3 (55–60%), whereas for IL-17A, CS3 and CS6 elicited the highest responder frequencies (40%).

No meaningful correlations were observed between maximal antigen-specific IFN-γ and IL-17A responses, although a weak correlation was detected for CS6 (Supplementary Table S1).

**Figure 3:**
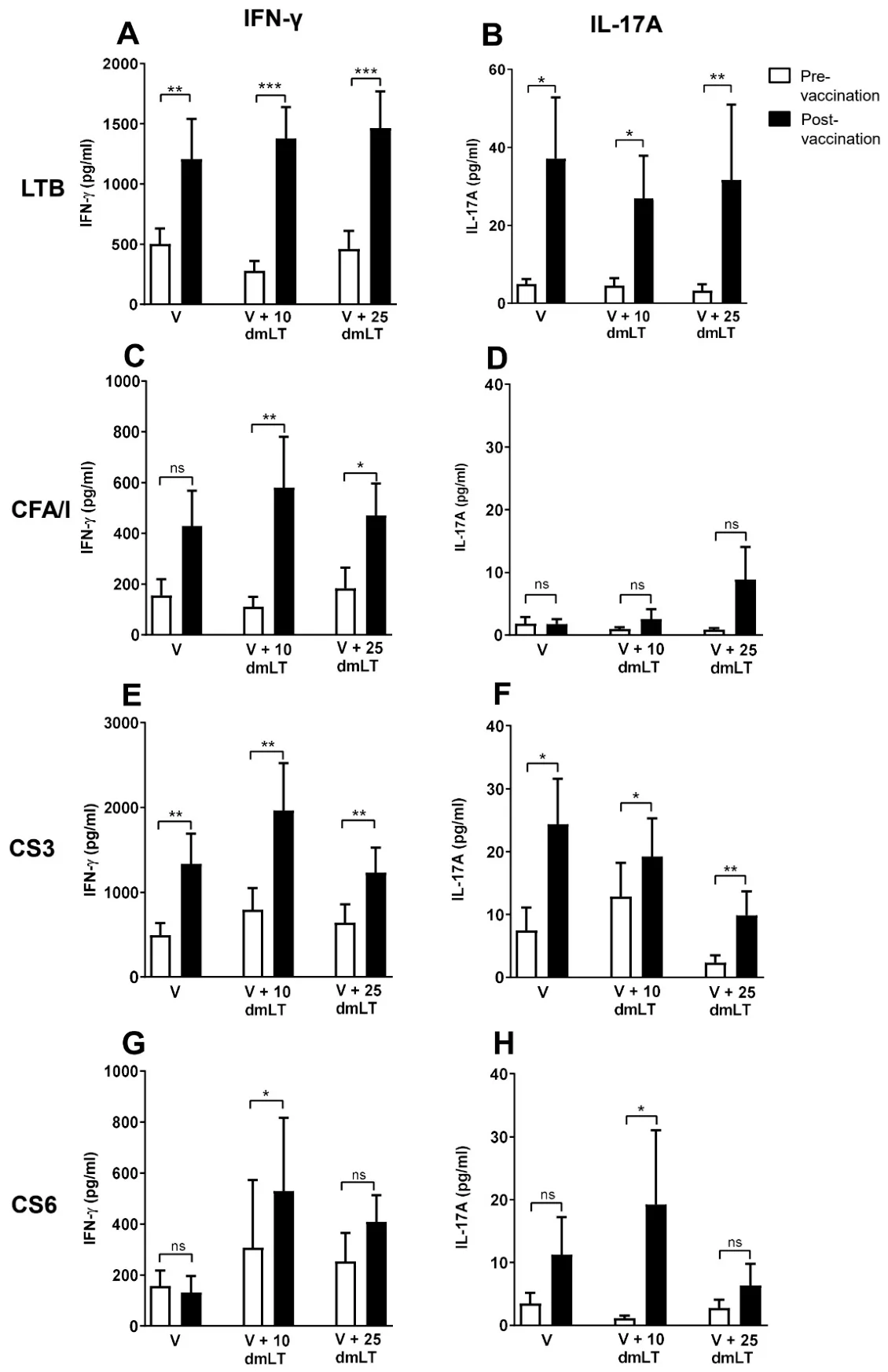
ETVAX-induced IFN-γ and IL-17A responses to LTB and colonization factors in the three vaccination groups. IFN-γ (**A**,**C**,**E**,**G**) and IL-17A (**B**,**D**,**F**,**H**) concentrations were measured in culture supernatants from PBMCs stimulated with LTB (**A**,**B**), CFA/I (**C**,**D**), CS3 (**E**,**F**) or CS6 (**G**,**H**) before and after vaccination. All subjects with available cryopreserved cells were included. Participants were stratified by vaccination group (*n* = 15–18 per group and antigen): vaccine alone (V), vaccine plus 10 μg dmLT (V + 10 dmLT), and vaccine plus 25 μg dmLT (V + 25 dmLT). Results are presented as mean + SEM of the maximal concentrations of cytokines detected at any post-vaccination time point (black bars) compared to pre-vaccination levels (white bars). Cytokine levels in unstimulated cultures were subtracted from all values shown. * *p* < 0.05, ** *p* < 0.01, *** *p* < 0.001; ns, *p* > 0.05; post- versus pre-vaccination.

Direct comparisons between vaccination groups revealed no statistically significant differences in cytokine response magnitudes for any antigen (*p* > 0.05, Figure 3). Nevertheless, several response patterns were observed. ETVAX induced significant IFN-γ and IL-17A responses to LTB and CS3 in all groups. Responses to CFA/I, CS5, and CS6 were generally weaker and less frequent in all groups. Significant IFN-γ responses to CFA/I were observed in both dmLT-adjuvanted groups, whereas significant IFN-γ responses to CS5 and CS6, and significant IL-17A responses to CS6, were observed only in the vaccine + 10 µg dmLT group.

Responder frequencies followed similar patterns in the different groups (Table 2). A tendency toward higher IFN-γ responder frequencies to LTB, CS5 and CS6 was observed in the vaccine + 10 µg dmLT group compared with vaccine alone; however, these differences were not statistically significant (*p* > 0.05). IL-17A responder frequencies were generally more comparable across groups.

Analysis of the T-cell response breadth showed that 50–70% of subjects responded with IFN-γ production to at least three antigens, and 20–40% to four or more antigens (Table 3). The vaccine + 10 µg dmLT group showed the broadest IFN-γ response profile, and a modest tendency toward broader combined IFN-γ and/or IL-17A responses than the other groups, although these differences were not statistically significant.

### 3.4. Relationship Between T-Cell and B-Cell Responses

To examine the relationship between cellular and humoral immunity, maximal IL-17A and IFN-γ responses (fold-rises) were compared with previously measured vaccine-specific IgA ALS responses from the same individuals [15]. Although similar antigen-recognition hierarchies were observed at the group level, no significant correlations were detected between individual antigen-specific ALS IgA and IFN-γ or IL-17A response magnitudes for any vaccine antigen (*p* > 0.05, Supplementary Table S2).

## 4. Discussion

T-cell responses induced by oral vaccines in humans remain incompletely characterized, yet they may provide important insights into the development of protective mucosal immunity and the mechanisms by which mucosal adjuvants modulate immune responses [9,41]. In this study we demonstrate that the oral ETEC vaccine candidate ETVAX induces IFN-γ and IL-17A responses directed against both the toxoid and CF components of the vaccine. IFN-γ levels were robust, whereas IL-17A responses were lower and required a highly sensitive ECL assay for consistent detection. The strongest IFN-γ and IL-17A responses were observed following stimulation with LTB and CS3, while responses to CFA/I, CS5, and CS6 were generally weaker. This T-cell response pattern mirrored IgA ALS responses, which were also strongest against LTB and CS3, likely influenced by the higher amounts of these antigens in the vaccine formulation [14,15].

Consistent with the findings from the present study, we have previously reported IFN-γ and IL-17A responses to LTB in a small group of individuals immunized with prototype ETEC vaccines containing LCTBA or the highly homologous CTB toxoid [22]. Previous studies of a first-generation ETEC vaccine consisting of inactivated natural ETEC strains expressing CFA/I, CS1, CS2, and CS3 in combination with CTB also demonstrated IFN-γ responses to CFA/I and CFA/II (comprising CS1 plus CS3), but did not detect IL-2 and did not assess other cytokines or CTB-specific responses [42]. The concurrent emergence of LTB- and CF-specific Th cells and ASCs in peripheral blood supports the potential of T-cell responses as complementary biomarkers of vaccine-induced mucosal immunity [9].

To investigate adjuvant effects, T-cell responses were also compared between ETVAX administered with and without 10 or 25 µg dmLT. While all T-cell analyses in this study were exploratory, the limited sample sizes make these between-group comparisons particularly hypothesis-generating and primarily intended for identifying broad trends rather than detecting modest differences. Within these limitations, it was notable that IFN-γ and IL-17A responses to CS6 reached statistical significance only in the group receiving ETVAX together with 10 µg dmLT. This pattern was consistent with IgA ALS results, in which significantly enhanced responses to CS6 were observed in the 10 µg dmLT group compared with vaccine alone, both in the total study cohort and in the subset included for T-cell analyses. We also observed a trend for increased breadth of IFN-γ responses when the vaccine was administered with 10 µg dmLT.

Both the cellular and ALS datasets suggested that responses to some of the lower-dose vaccine antigens were more pronounced in recipients of 10 than 25 µg dmLT. Al-though the underlying mechanisms remain poorly defined, similar non-linear dose–response relationships have been reported for dmLT and related LT-derived adjuvants in both animal and human studies [27,43,44], suggesting that adjuvant activity may reach a plateau beyond which increasing the dose provides limited benefit. The concordant trends across cellular and mucosal immune readouts raise the possibility that dmLT may be particularly effective at augmenting responses to less abundant or less immunogenic vaccine antigens. This interpretation is consistent with dose-sparing effects of dmLT on CF responses to a prototype ETVAX vaccine previously reported in a mouse model [14]. Such effects are particularly relevant for pediatric immunization, where reduced ETVAX doses are used to minimize vomiting [19]. Studies in Bangladesh have demonstrated that ETVAX is highly immunogenic in children and infants, with dmLT enhancing fecal and plasma IgA responses to fractionated doses [19,45]. More recent studies performed in The Gambia and Zambia further support the feasibility and immunogenicity of administering lower doses of ETVAX with dmLT in infants and young children [12,13].

In parallel with these clinical observations, experimental in vitro studies have provided insights into the immunomodulatory mechanisms through which dmLT may enhance vaccine-induced immune responses. We have previously shown that dmLT potently enhances IL-17A production in PBMCs stimulated with mitogens, superantigens, and a range of non-ETEC vaccine antigens, whereas effects on IFN-γ responses were more variable [21,22,24]. Nevertheless, both IL-17A and IFN-γ responses to LTB were enhanced by addition of dmLT in PBMC cultures derived from individuals immunized with prototype ETEC vaccines containing LCTBA or CTB [22]. Mechanistically, we have demonstrated that the IL-17A-enhancing effect of dmLT is dependent on IL-1β production and cAMP signaling in APCs, whereas the pathways underlying its effects on IFN-γ production remain to be fully elucidated [21,24]. Notably, the in vitro enhancing effects of dmLT on PBMC cytokine responses were also more pronounced when cells were stimulated with lower concentrations of whole-cell ETVAX component or purified *E. coli* LPS [24].

dmLT has been reported to improve the protective efficacy of a live ETEC vaccine in adults, but the immunological mechanisms underlying this effect remain to be defined [46]. In murine models, dmLT strongly enhances IL-17A responses to several mucosal vaccines, including those targeting *Helicobacter* and pneumococcal infections [16,25,26,27]. Additionally, dmLT enhances both mucosal and systemic IgA responses to all major components of ETVAX in mice [14]. A related LT-derived adjuvant, mLT, which contains only one of the two mutations present in dmLT, has been shown to enhance IFN-γ responses to a *Campylobacter* vaccine in humans, an effect that correlated with increased resistance to subsequent experimental *Campylobacter* challenge [47,48].

The functional roles of IL-17A and IFN-γ in immunity to ETEC likely vary according to the anatomical site of cytokine production. Proposed protective mechanisms include enhanced antigen presentation (IFN-γ), recruitment of immune cells to the gut (IFN-γ, IL-17A), promotion of germinal center formation (IL-17A), induction of IgA responses (IL-17A) and upregulation of epithelial polymeric Ig receptor expression (IL-17A), leading to increased transportation of SIgA into the intestinal lumen [41,49,50]. Increased IFN-γ has been observed in the intestinal mucosa following oral immunization with the CTB-containing cholera vaccine Dukoral, supporting intestinal migration of Th1 cells [36].

Notably, the Th responses induced by ETVAX vaccination resemble those observed following ETEC infection. We have previously shown that ETEC infection in Bangladeshi adults predominantly induces Th17 responses, accompanied by Th1 responses [51]. Similarly, participants in a controlled human ETEC challenge study exhibited increased proportions of circulating Th17-like cells, expressing gut-homing markers, with early induction associated with protection against diarrhea [52]. In addition to these cellular responses, both natural infection and ETVAX vaccination induce memory B-cell responses to LTB and CFs [17,53], which are likely to be dependent on Th cells for their induction. Given that natural ETEC infection is associated with protection against homologous reinfection [1,13,54], the induction of similar Th response profiles by ETVAX supports the notion that the vaccine engages immunological pathways relevant for protective immunity.

Although cytokine and IgA responses showed similar antigen-recognition hierarchies at the group level, no correlations between response magnitudes were observed at the individual level. The substantial inter-individual variability in cytokine response kinetics may partly explain this lack of correlation. In contrast to the T-cell responses, ALS responses following immunization with ETVAX or the CTB-containing oral cholera vaccine Dukoral more consistently peak on day 5 after the second dose in the vast majority of individuals [6,15,39]. Moreover, cytokine production following antigen stimulation represents only one aspect of T-cell function and may not directly reflect the quality or magnitude of T-cell help provided to B cells. Importantly, we previously found that the frequency of activated ICOS+ CD4+CXCR5+ cTfh cells correlated with both primary ALS responses and memory B-cell responses measured 1–2 years later in the same study cohort [20]. cTfh-like cells are mobilized into peripheral blood following vaccination and are thought to reflect ongoing germinal center activity [55,56,57]. We showed that the ETVAX-induced activated cTfh cells expressed gut-homing and Th17-associated markers and promoted LTB-specific IgA production from cocultured B cells isolated from ETVAX vaccinees [20]. However, owing to limited cell availability, direct characterization of the cytokine production of antigen-specific cTfh cells was not feasible. Although cTfh cells can produce cytokines associated with Th1, Th17, and Th2 subsets and Th17-like cTfh cells have been described as particularly efficient B-cell helpers, they are unlikely to be the major source of cytokines measured in our PBMC cultures as they represent only ~10% of circulating CD4^+^ T cells and generally produce relatively low cytokine levels [20,55,58]. The IFN-γ and IL-17A detected in our cultures were more likely derived from central or effector memory Th cell populations. Future studies should include comprehensive analyses of antigen-specific cTfh and other Th subsets across multiple time points to capture the full response dynamics following both oral vaccination and natural ETEC infection.

Strengths of this study include the integrated analysis of both antigen-specific IgA ALS and T-cell responses in the same subjects across multiple time points, as well as the inclusion of groups vaccinated both with and without dmLT. This represents one of the few studies to characterize antigen-specific T-cell responses to an oral ETEC vaccine in humans and, to our knowledge, the largest and most detailed analyses comparing T-cell responses to multiple ETEC antigens. The availability of matching cellular and intestine-derived ALS data enabled integrated analyses of vaccine-induced immune responses.

Several limitations should also be acknowledged. The number of subjects with sufficient numbers of cryopreserved PBMCs available for cellular analyses was relatively small, particularly within individual vaccination groups, limiting statistical power for between-group comparisons. However, comprehensive cellular analyses in human oral vaccine trials are technically and practically demanding and therefore rarely conducted in large cohorts. Consequently, despite its moderate size, the present study expands the limited knowledge regarding T-cell responses to oral vaccination in humans. The paired pre- and post-vaccination design allowed each participant to serve as their own control; however, inclusion of placebo recipients in the T-cell analyses would have provided additional support for attributing the observed responses specifically to vaccination. The analyzed subset was broadly representative of the original study population with respect to vaccine-induced IgA ALS responses, although some differences were observed compared with the full cohort. The initial cytokine screening was restricted to participants with strong vaccine-specific IgA responses, which facilitated assay development, but may have limited the identification of less prominent cytokine signatures in the larger study population.

In addition, the current study focused on antigen-induced cytokine production and did not include flow cytometric characterization of responding T-cell subsets. Therefore, the cellular sources of the observed cytokine responses could not be directly defined, although depletion experiments supported a major contribution by CD4+ T cells. The cytokine findings complement our previous characterization of cTfh responses in the same study population. While the supernatant assay provides less phenotypic resolution than flow cytometry-based approaches, its relative simplicity may facilitate standardization across laboratories. Future studies incorporating complementary analytical platforms and larger, independent cohorts will be important to confirm and extend our findings.

In conclusion, oral ETVAX vaccination induced antigen-specific IFN-γ and IL-17A responses to both LTB and CF antigens. T-cell response patterns broadly paralleled antigen-specific IgA ALS responses across vaccine antigens, supporting a role for cellular immunity in mucosal vaccine responses. Similar patterns in relation to dmLT were observed in both the cellular and humoral datasets. Together, these findings provide new insight into the cellular immunogenicity of ETVAX and highlight the value of integrating antigen-specific T-cell and antibody measurements in the evaluation of oral vaccines.

## Figures and Tables

**Table 1:** Demographics of the total study population ^a^ and the T-cell analysis subset.

	All Vaccinees		Vaccine Groups					
			Vaccine		Vaccine + 10 µg dmLT		Vaccine + 25 µg dmLT	
	Total ^a^ *n* = 84	Subset *n* = 52	Total *n* = 29	Subset *n* = 17	Total *n* = 29	Subset *n* = 17	Total *n* = 26	Subset *n* = 18
Age, median years (range)	24 (18–43)	24 (18–43)	24 (20–39)	24 (21–32)	24 (20–43)	25 (21–43)	24 (18–30)	24 (18–30)
Sex, *n* (%)								
Males	44 (52%)	30 (58%)	13 (45%)	6 (35%)	12 (41%)	8 (47%)	19 (73%)	16 (89%)
Females	40 (48%)	22 (42%)	16 (55%)	11 (65%)	17 (59%)	9 (53%)	7 (27%)	2 (11%)

^a^ The total study population included in the per-protocol set evaluated for IgA responses in the antibody in lymphocyte supernatants (ALS) assay in the original study [15].

**Table 2:** Frequencies of IFN-γ and IL-17A responders ^a^ against the different primary vaccine antigens.

	All Vaccinees in T-Cell Subset	Vaccine Groups		
		Vaccine	Vaccine + 10 µg dmLT	Vaccine + 25 µg dmLT
IFN-γ				
LTB	37/52 (71%)	10/17 (59%)	14/17 (82%)	13/18 (72%)
CFA/I	29/49 (59%)	9/16 (56%)	9/15 (60%)	11/18 (61%)
CS3	28/51 (55%)	10/17 (59%)	10/16 (63%)	8/18 (44%)
CS5	21/47 (45%)	7/15 (47%)	9/15 (60%)	5/17 (29%)
CS6	15/47 (32%)	2/15 (13%)	5/15 (33%)	8/17 (47%)
IL-17A				
LTB	30/52 (58%)	9/17 (53%)	10/17 (59%)	11/18 (61%)
CFA/I	11/49 (22%)	2/16 (13%)	5/15 (33%)	4/18 (22%)
CS3	21/51 (41%)	8/17 (47%)	6/16 (38%)	7/18 (39%)
CS5	nd	nd	nd	nd
CS6	18/47 (38%)	7/15 (47%)	8/15 (53%)	3/17 (18%)

^a^ Cumulative response rates after one or two immunizations. Responders were defined as subjects showing a ≥2-fold increase in cytokine concentrations post-vaccination compared with pre-vaccination. *p* > 0.05 for all comparisons of response rates in subjects receiving vaccine alone or with dmLT 10 or 25 µg/mL. nd; not determined.

**Table 3:** Frequencies of IFN-γ and/or IL-17A responders against different numbers of ETVAX vaccine antigens.

	IFN-γ Responses ^a^			IL-17A Responses ^b^			IFN-γ and/or IL-17A Responses ^b^		
Frequency of subjects responding to:	**Vaccine**	**Vaccine** **+ 10 µg** **dmLT**	**Vaccine** **+ 25 µg** **dmLT**	**Vaccine**	**Vaccine** **+ 10 µg dmLT**	**Vaccine** **+ 25 µg dmLT**	**Vaccine**	**Vaccine** **+ 10 µg dmLT**	**Vaccine** **+ 25 µg dmLT**
5 antigens	13%	20%	18%	nd	nd	nd	nd	nd	nd
≥4 antigens	20%	40%	29%	0%	7%	6%	33%	40%	31%
≥3 antigens	47%	67%	53%	40%	40%	25%	67%	73%	63%
≥2 antigens	60%	80%	65%	50%	60%	44%	67%	87%	75%
≥1 antigen	93%	100%	82%	75%	80%	69%	93%	100%	88%

^a^ Cumulative response rates to 5 antigens (LTB, CFA/I, CS3, CS5, CS6) after one or two immunizations. ^b^ Cumulative response rates to 4 antigens (LTB, CFA/I, CS3, CS6) after one or two immunizations. Vaccine, *n* = 15, Vaccine + 10 µg dmLT, *n* = 15; vaccine + 25 µg dmLT, *n* = 17. nd; not determined.

## Data Availability

The data presented in this study are available on request from the corresponding author due to anonymized data.

## Supplementary Materials

The following supporting information can be downloaded at https://www.mdpi.com/article/10.3390/microorganisms14092007/s1. Table S1: Correlation between fold-increases in IFN-γ and IL-17A concentrations elicited by stimulation of PBMCs with different ETVAX vaccine antigens; Table S2: Correlation between fold-increases in levels of T helper cell-associated cytokines and IgA antibody in lymphocyte secretions (ALS) directed against ETVAX vaccine antigens; Figure S1: IgA ALS responses to LTB and colonization factors in the original study population and T-cell analysis subset; Figure S2: IgA ALS responses to LTB and colonization factors in the original study population and T-cell analysis subset in the different vaccination groups.

## Abbreviations

ALS, antibodies in lymphocyte supernatant; APC, antigen presenting cell; ASC, antibody-secreting cell; CFs, colonization factors; CT, cholera toxin; CTB, cholera toxin B-subunit; cTfh, circulating T follicular helper-like cell; dmLT, double mutant heat labile toxin; ECL, electrochemiluminescence; ETEC, enterotoxigenic *Escherichia coli*; LCTBA, CTB/LTB hybrid protein; LT, heat-labile toxin; LTB, heat-labile toxin B-subunit; mLT, single mutant LT; mmCT, multiple mutated CT; PBMCs, peripheral blood mononuclear cells; SIgA; secretory IgA; Tfh, T follicular helper cell; Th, T helper cell.
