# Supplementary material for: Th1 and Th17 Responses to LTB and Colonization Factors Following Oral ETEC Vaccination
Source: Microorganisms. 2026 Sep 10;14(9):2007. doi: 10.3390/microorganisms14092007 (PMC13609440; doi:10.3390/microorganisms14092007)
Supplement: Supplementary file 1 [file microorganisms-14-02007-s001.zip › microorganisms-4389849-supplementary.pdf]

Supplementary tables

Supplementary Table S1. Correlation between fold-increases in IFN-γ and IL-17A concentrations elicited by stimulation of PBMCs with different ETVAX vaccine antigens.

	IFN-γ vs. IL-17A	
**Antigen**	**Spearman r**	**P-value**
LTB	0.20	0.16
CFA/I	0.24	0.09
CS3	0.27	0.05
CS5	nd	nd
CS6	0.34	0.02

nd; not determined

Supplementary Table S2. Correlation between fold-increases in levels of T helper cell-associated cytokines and IgA antibody in lymphocyte secretions (ALS) directed against ETVAX vaccine antigens.

	IFN-γ vs. ALS		IL-17A vs. ALS	
**Antigen**	**Spearman r**	**P-value**	**Spearman r**	**P-value**
LTB	-0.04	0.79	-0.03	0.84
CFA/I	0.14	0.34	0.29	0.05
CS3	0.04	0.77	-0.02	0.87
CS5	-0.15	0.33	nd	nd
CS6	0.09	0.55	0.11	0.47

nd; not determined

Supplementary figures

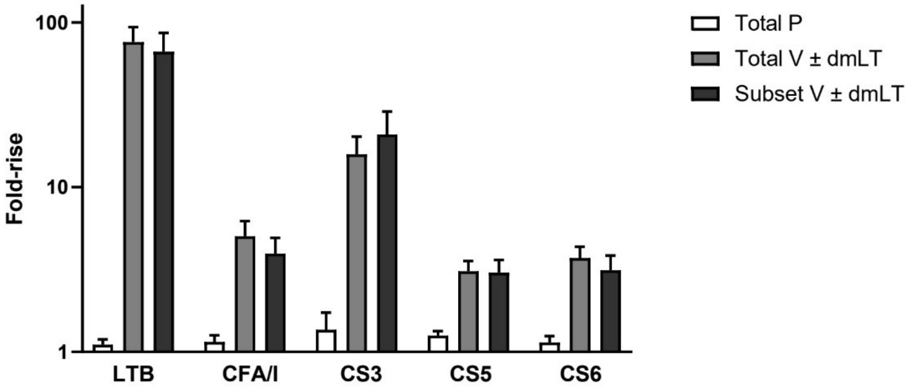

Supplementary Figure S1. IgA ALS responses to LTB and colonization factors in the original study population and T-cell analysis subset. ALS data from the original study population have been reported previously per vaccination group [Supplementary reference 1]. Here, these data are presented as a pooled cohort to assess the representativeness of the subset included in the T-cell analyses. For each subject, the maximal post-vaccination fold-rise in antibody levels against each antigen (LTB, CFA/I, CS3, CS5, and CS6) relative to the levels in the pre-vaccination sample was calculated across all post-vaccination time points. The total study population comprised all participants evaluated for ALS responses in the original study, including placebo recipients (Total P, white bars, n = 24-29) and participants from all three vaccine groups combined (Total V ± dmLT, grey bars, n = 76-84). Responses in the T-cell analysis subset are shown by black bars (Subset V ± dmLT, n = 47–52). Data are presented as geometric mean fold-rises + SEM.

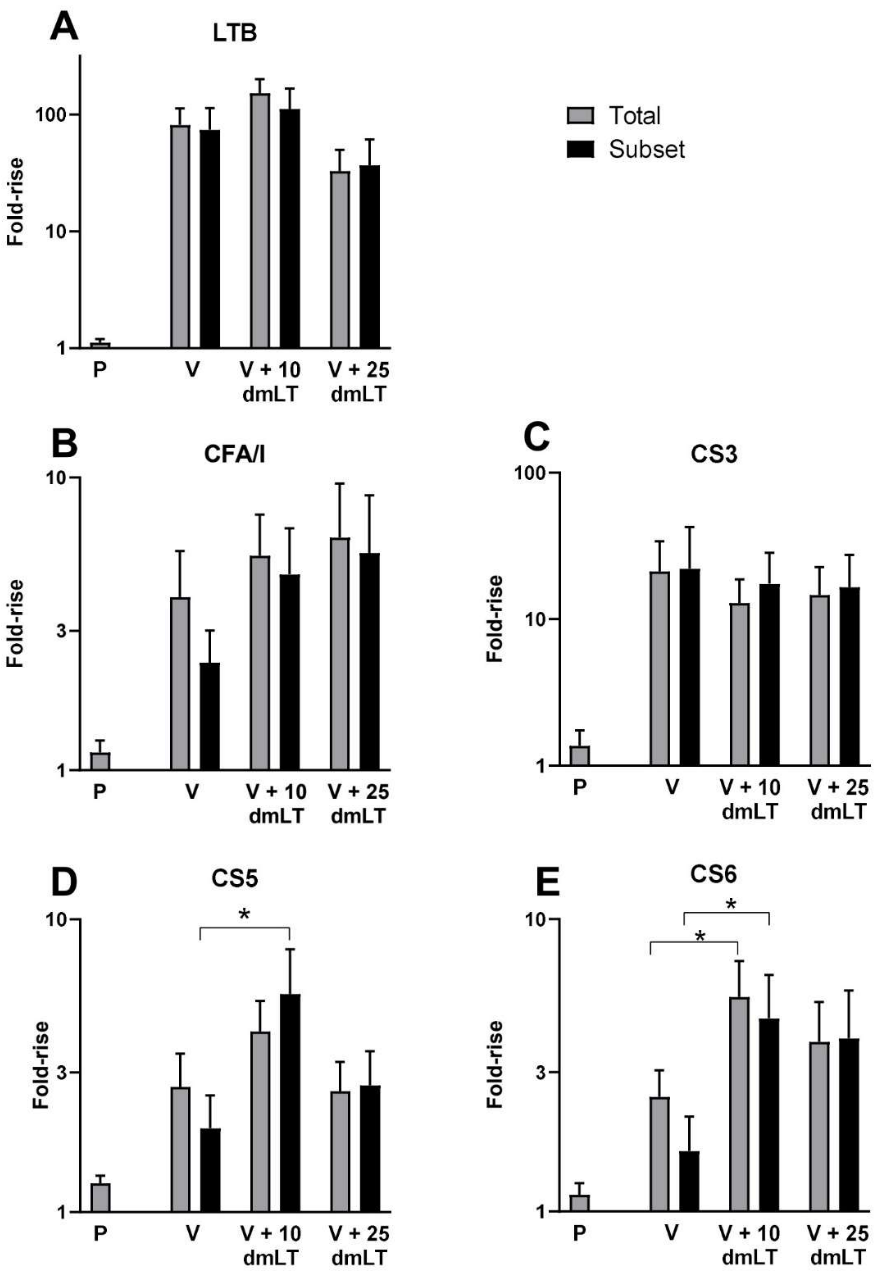

Supplementary Figure S2. IgA ALS responses to LTB and colonization factors in the original study population and T-cell analysis subset in the different vaccination groups. ALS data for the original study population were previously reported [Supplementary reference 1] and are reproduced here to assess the representativeness of the T-cell analysis subset. For each participant, the maximal post-vaccination fold-rise in antibody levels against each antigen (LTB, CFA/I, CS3, CS5, and CS6) relative to baseline was calculated across all post-vaccination time points. Participants were stratified by vaccination group: placebo (P), vaccine alone (V), vaccine plus 10 µg dmLT (V + 10 dmLT), and vaccine plus 25 µg dmLT (V + 25 dmLT). Responses are shown for the total ALS analysis population (Total, grey bars) and the T-cell analysis subset (Subset, black bars). Data are presented as geometric mean fold-rises + SEM. * $P < 0.05$ after Holm's correction for multiple testing and in the unadjusted analysis (vaccine + dmLT groups versus vaccine alone).

Supplementary reference

1. Lundgren, A.; Bourgeois, L.; Carlin, N.; Clements, J.; Gustafsson, B.; Hartford, M.; Holmgren, J.; Petzold, M.; Walker, R.; Svennerholm, A.M. Safety and immunogenicity of an improved oral inactivated multivalent enterotoxigenic *Escherichia coli* (ETEC) vaccine administered alone and together with dmlt adjuvant in a double-blind, randomized, placebo-controlled phase i study. *Vaccine* **2014**, *32*, 7077-7084.
